# A Design Method and Application of Meta-Surface-Based Arbitrary Passband Filter for Terahertz Communication

**DOI:** 10.3390/s24041291

**Published:** 2024-02-17

**Authors:** Da Hou, Lihui Wang, Qiuhua Lin, Xiaodong Xu, Yin Li, Zhiyong Luo, Hao Chen

**Affiliations:** 1 School of Electronics and Communication Engineering, Sun Yat-sen University, Shenzhen 518107, China; houd5@mail2.sysu.edu.cn (D.H.); wanglh65@mail2.sysu.edu.cn (L.W.); linqh26@mail2.sysu.edu.cn (Q.L.); 2 Pengcheng Laboratory, Shenzhen 518107, China; xuxiaodong@bupt.edu.cn (X.X.); liy17@pcl.ac.cn (Y.L.); 3 School of Information and Communication Engineering, Beijing University of Posts and Telecommunications, Beijing 100876, China; 4 Shenzhen Key Laboratory of Navigation and Communication Integration, Shenzhen 518107, China

**Keywords:** terahertz, silicon substrate integrated gap waveguide (SSIGW), through-silicon via (TSV), meta-surface, filter

## Abstract

A meta-surface-based arbitrary bandwidth filter realization method for terahertz (THz) future communications is presented. The approach involves integrating a meta-surface-based bandstop filter into an ultra-wideband (UWB) bandpass filter and adjusting the operating frequency range of the meta-surface bandstop filter to realize the design of arbitrary bandwidth filters. It effectively addresses the complexity of designing traditional arbitrary bandwidth filters and the challenges in achieving impedance matching. To underscore its practicality, the paper employs silicon substrate integrated gap waveguide (SSIGW) and this method to craft a THz filter. To begin, design equations for electromagnetic band gap (EBG) structures were developed in accordance with the requirements of through-silicon via (TSV) and applied to the design of the SSIGW. Subsequently, this article employs equivalent transmission line models and equivalent circuits to conduct theoretical analyses for both the UWB passband and the meta-surface stopband portions. The proposed THz filter boasts a center frequency of 0.151 THz, a relative bandwidth of 6.9%, insertion loss below 0.68 dB, and stopbands exceeding 20 GHz in both upper and lower ranges. The in-band group delay is 0.119 ± 0.048 ns. Compared to reported THz filters, the SSIGW filter boasts advantages such as low loss and minimal delay, making it even more suitable for future wireless communication.

## 1. Introduction

The “International Mobile Telecommunications (IMT) Framework and General Objectives Proposal for 2030 and Future Development” program document’s completion in 2023 ushers in a new phase of future wireless communications development. Terahertz (THz) technology, one of the primary future wireless communication technologies, will be extensively used in both the civil and military sectors. As an indispensable component in communication systems, filters play a crucial role in the efficient and low-loss design of the THz band. However, THz filters, recognized as pivotal components, currently grapple with challenges such as significant losses, integration complexities, and difficulties in achieving arbitrary bandwidth designs. The current implementation of THz filters relies mainly on meta-material design [1,2], making it challenging to integrate them into silicon-based front-end circuits. Some waveguide-based filters’ [3,4] design faces issues such as surface waves and high interconnection losses.

In 2015, substrate integrated gap waveguide (SIGW) was proposed to solve the problems of loss and packaging in the design of high-frequency devices [5]. SIGW has the capability to mitigate surface waves and space radiation in transmission lines, providing a favorable environment for millimeter-wave (MMW) circuits and their packaging [6,7,8]. However, when SIGW is applied in the THz range, the waveguide structures become extremely small, making them challenging to manufacture. Additionally, issues such as high losses and difficulties in integration arise. Fortunately, through-silicon via (TSV), a key semiconductor technology of 3D integrated circuits, has high-precision trench etching and metal filling on the order of microns and very low losses, which has aroused significant research interest [9]. Moreover, TSV can be connected to other planar devices through a redistribution layer [10]. Therefore, those attractive features have made TSV technology a good alternative for miniaturization and integration of passive devices. Indeed, some passive devices based on TSV have been proposed [3,11]. Recently, due to the large-scale expansion of semiconductor technology and emerging markets such as automotive radar and 5G, TSV technology has been gradually extended to MMW and THz fields [12,13,14]. In 2022, a silicon substrate integrated gap waveguide (SSIGW) utilizing TSV was proposed to address the aforementioned issues, and it has been successfully applied in the design of a 6G filtering metalens antenna [15]. Therefore, SSIGW, characterized by low losses, compact dimensions, suppression of surface waves, and ease of integration, emerges as a superior alternative for THz circuit design.

**Figure 1:**
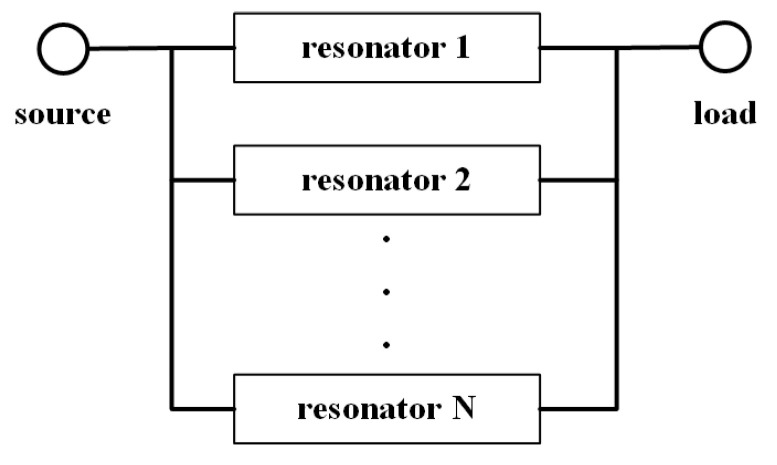
Diagram of the traditional implementation principle of arbitrary bandwidth filters.

Additionally, regarding filter design, it is common for designs to target specific frequency bands and ranges, with fixed structures, making it challenging to achieve arbitrary bandwidths. Currently, only a few studies have addressed this issue [16,17], all adopting the approach of increasing resonance points, as illustrated in Figure 1, which depicts the basic principle. One study proposed a filter prototype synthesis method for designing filters with arbitrary bandwidths [16]. Another study presented a method based on the theory of small reflections to design microstrip filters with arbitrary passbands [17]. It introduced a novel stub-loaded microstrip filter and derived an approximate reflection coefficient function for the input of the filter. However, these methods face difficulties in computation and complexities in design. Due to its simplicity in design and controllable parameters, electromagnetic meta-surfaces have been widely used in device design [18,19]. Based on the meta-surface resonance theory, this paper proposes a method of utilizing meta-surfaces in the design of ultra-wideband (UWB) filters, offering a solution for achieving filters with arbitrary bandwidths. And the proposed method offers advantages such as simplicity in implementation and high flexibility.

This work proposes a method for the design of meta-surface filters with arbitrary passband, and for verification, a filter based on SSIGW is presented. In Section 2, the method for the design of meta-surface filters with arbitrary passband is given. Furthermore, an SSIGW meta-surface filter based on the design method has been proposed. In Section 3, a comprehensive analysis of the proposed filter has been undertaken, covering the theoretical design of SSIGW, the design of the UWB filter, and the meta-surface-based wideband bandstop filter design. Comparative evaluations with relevant THz filters have highlighted its superior performance. In Section 4, some conclusions are given.

## 2. Meta-Surface-Based Arbitrary Bandwidth SSIGW Filter Design Method

**Figure 2:**
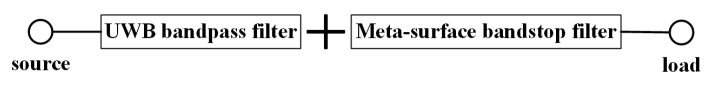
The proposed diagram for the implementation principle of arbitrary bandwidth filters.

**Figure 3:**
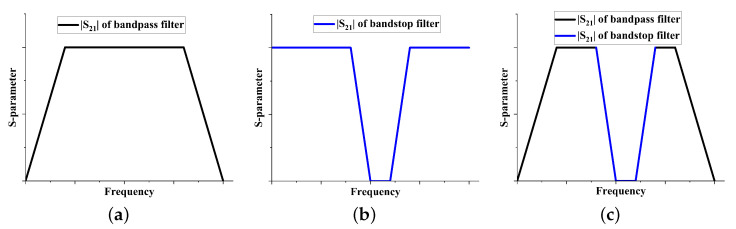
Theoretical performance of arbitrary bandwidth filter: (**a**) bandpass filter; (**b**) bandstop filter; and (**c**) required filter.

This section presents a method for designing an arbitrary bandwidth SSIGW filter based on meta-surfaces. The principle of the proposed method is illustrated in Figure 2. It entails incorporating a meta-surface-based bandstop filter into a UWB bandpass filter, with the controllable filter bandwidth achieved through the adjustment of the meta-surface’s dimensions. The decision to employ meta-surface design for the bandstop structure is motivated by the challenges and complexity associated with achieving designs for diverse frequency bands using traditional bandstop filter structures. Furthermore, achieving perfect impedance matching with UWB filter structures has proven difficult using conventional band-stop filter configurations. Meta-surfaces offer advantages such as structural simplicity and flexibility, enabling the flexible design of filters with varying bandwidths and stopband widths to meet specific requirements. Figure 3 illustrates the theoretical performance of the proposed method. In Figure 3a, the performance of the bandpass filter is shown, and in Figure 3b, the performance of the bandstop filter is depicted. By superimposing these two, the desired filter can be obtained, as shown in Figure 3c.

**Figure 4:**
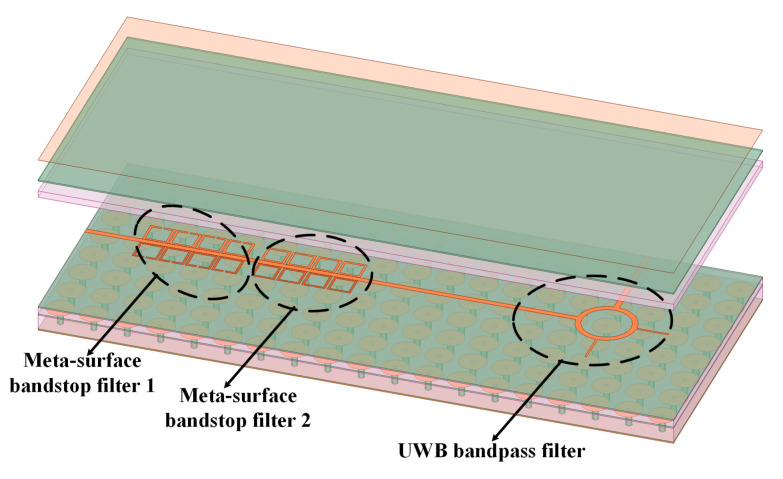
The physical structure of the proposed filter.

To validate the efficacy of the proposed approach, an SSIGW THz filter was designed employing split-ring resonator (SRR) meta-surfaces, as depicted in the overall structure illustrated in the Figure 4. It comprises two components: a UWB bandpass filter and a meta-surface wideband bandstop filter. Additionally, the meta-surface wideband bandstop filter is composed of meta-surface bandstop filter 1 and meta-surface bandstop filter 2. To provide a clearer exposition of the design concept, the subsequent sections will individually analyze the design of SSIGW, UWB filters, and meta-surface-based wideband bandstop filters.

## 3. Physical Realization of Arbitrary Bandwidth SSIGW Filter Based on Meta-Surface

According to the literature [20], the sixth-generation mobile communication technology (6G) band includes the G band (0.14–0.22 THz). Therefore, this paper presents the detailed design of the filter in this frequency band, aiming to offer new insights into the filter design for 6G communication and promote the development of 6G communication. The concrete implementation of the proposed arbitrary bandwidth SSIGW filter based on meta-surface encompasses three key components: the theoretical design of SSIGW, the design of the ultra-wideband filter, and the design of the meta-surface-based bandstop filter with a wide stopband. The following subsections will delve into each of these aspects individually.

### 3.1. Design of SSIGW

Beginning with TSV technology, this subsection researches the design of SSIGW for the THz band. The dielectric constants of the materials employed in all structures within this artical are Si with $\varepsilon_{s}=11.9$, and SiO_2_ with $\varepsilon_{o}=4$.

**Figure 5:**
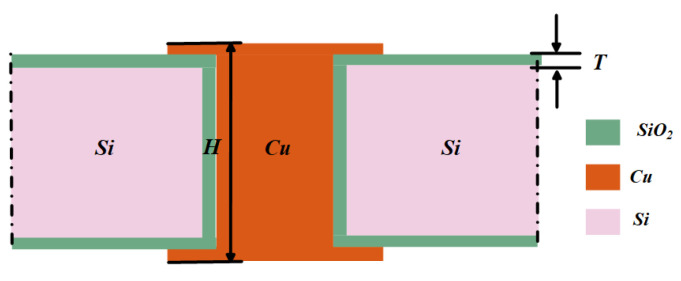
Schematic diagrams of a TSV cell.

As per the information presented in [15], SSIGW is composed of a TSV-based electromagnetic band gap (EBG) array structure and a stripline. In this subsection, the TSV-based EBG structure is first given and analyzed so that an SSIGW can work for the THz band. TSV is formed in a silicon substrate with limited and nonzero resistivity and has a metal–insulator–semiconductor (MIS) structure [21], as shown in Figure 5, i.e., metal (copper), insulator (SiO_2_), and semiconductor substrate (Si) between signal and ground [22]. The thickness of the insulator layer (*T*) is determined by the following formulae:

(1) $$T>r\left(1-e^{-\frac{2\pi \varepsilon_{o}H}{\mathrm{Cox}(f)}}\right)$$

and

(2) T>0.2μm

in which *T* is the thickness of the insulator SiO_2_ layer outside the copper/silicon pillar; *r* is the radius of the copper column; *H* is the height of the copper column; and Cox(f) is the maximum capacitance of the sidewall, which can be obtained by simulation.

**Figure 6:**
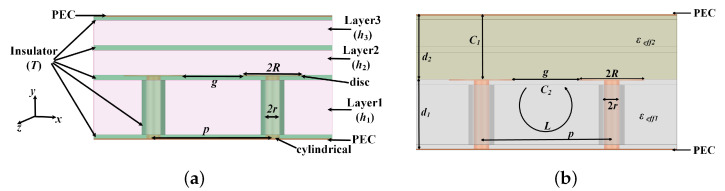
Side view of TSV-based EBGs: (**a**) physical structure and (**b**) equivalent physical structure.

Figure 6a shows the side view of TSV-based EBGs with indicated parameters, such as dimensions $d_{2}$, *R*, *p*, *r*, *T*, and *g*. Due to the computational difficulty of embedding the TSV-based EBG in two media, for simplicity, we assume that the TSV-based EBG is embedded in a medium with an equivalent relative permittivity, as shown in Figure 6b. Figure 6b shows the side view of TSV-based EBGs’ equivalent structure with indicated parameters, such as capacitance $C_{1}$ and $C_{2}$, inductance *L*, and dimensions $d_{1}=h_{1}+2T$, $d_{2}=h_{2}+h_{3}+2T$. The effective relative dielectric constants $\varepsilon_{eff1}$ and $\varepsilon_{eff2}$ are obtained from (3) and (4) using the weighted average method.

(3) $$\begin{array}{c} \varepsilon_{eff1}=\frac{2T\varepsilon_{o}+\left(p^{2}-\pi T^{2}\right)h_{1}\varepsilon_{s}/p^{2}}{d_{1}}+\frac{\pi T^{2}h_{1}\varepsilon_{o}/p^{2}}{d_{1}} \end{array}$$

(4) $$\varepsilon_{eff2}=\frac{2T\varepsilon_{o}+\left(h_{2}+h_{3}\right)\varepsilon_{s}}{d_{2}}$$

Applying the theory in [23,24] to Figure 6b, the following formulae can be obtained.

(5) $$C_{1}=\varepsilon_{0}\varepsilon_{eff2}\pi R^{2}/d_{1}$$

(6) $$C_{2}=4\varepsilon_{0}\varepsilon_{eff1}\pi R/\log\left(\sec\left(\pi \left(p-2R\right)/2p\right)\right)$$

(7) $$L=-\mu_{0}d_{1}\left(4+\ln\left(p-2r\right)\right)/2\pi$$

(8) $$f_{0}=1/2\pi \sqrt{LC_{1}C_{2}/\left(C_{1}+C_{2}\right)}$$

(9) $$FBW=\sqrt{L\left(C_{1}+C_{2}\right)/C_{1}C_{2}}/\eta$$

where the vacuum dielectric constant is $\varepsilon_{0}=8.854\times 10^{-12}$ F/m, vacuum permeability is $\mu_{0}=4\pi \times 10^{-7}$ H/m, effective impedance is η, FBW is the fractional bandwidth around the center frequency $f_{0}$, *p* is the permutation period between the TSV-based EBGs, *r* is the radius of the column, *R* is the radius of the disc, $d_{1}$ is the height of the column, $d_{1}$ is the thickness of the dielectric plate above the disc, $C_{1}$ is the capacitance of the disc with the top PEC, $C_{2}$ is the gap capacitance between the two discs, and *L* is the inductance of the copper column.

Firstly, it is assumed that the height of the copper column is $d_{1}$ = 65 μm, the thickness of the medium plate above the copper column is $d_{2}$ = 60 μm, the center frequency is $f_{0}$ = 0.19 THz, and the fractional bandwidth is FBW = 65%. Additionally, according to formulae (5)–(9), we obtain *R* = 47.8 μm and *r* = 6.6 μm. Lastly, for convenience of the design, set *R* = 50 μm and *r* = 7 μm.

According to [14], it can be known that Cox(f) tends to 0 at the frequency infinity. This work considers the THz band, which suggests Cox(f) = 0.03 pF, and thus according to formula (1), *T* > 3.5 μm is obtained. Therefore, set *T* = 5 μm.

**Figure 7:**
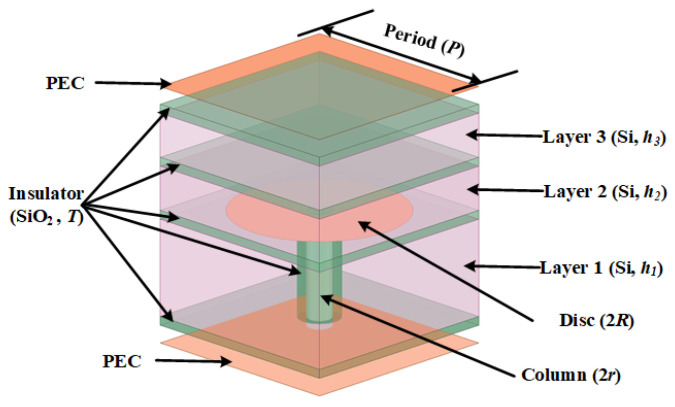
TSV-based EBG cell structure.

The designed TSV-based EBG structure unit is shown in Figure 7. The upper surface of the dielectric substrate (Layer 1) is coated with a layer of SiO_2_. A metal disc with a diameter of 2*R* is printed on the upper surface of the insulator. And the lower surface of Layer 1 is also coated with a layer of SiO_2_. A copper column with a diameter of 2*r* is inserted into the substrate, and a layer of SiO_2_ with a thickness of T is wrapped outside the copper pillar. The upper surface of the middle dielectric substrate (Layer 2, Si) is covered with a layer of SiO_2_. The upper surface of the upper dielectric substrate (Layer 3, Si) is also coated with a layer of SiO_2_, and on the upper surface, there is a copper-clad metal layer. The dimensions of the TSV-based EBG are listed in Table 1.

**Figure 8:**
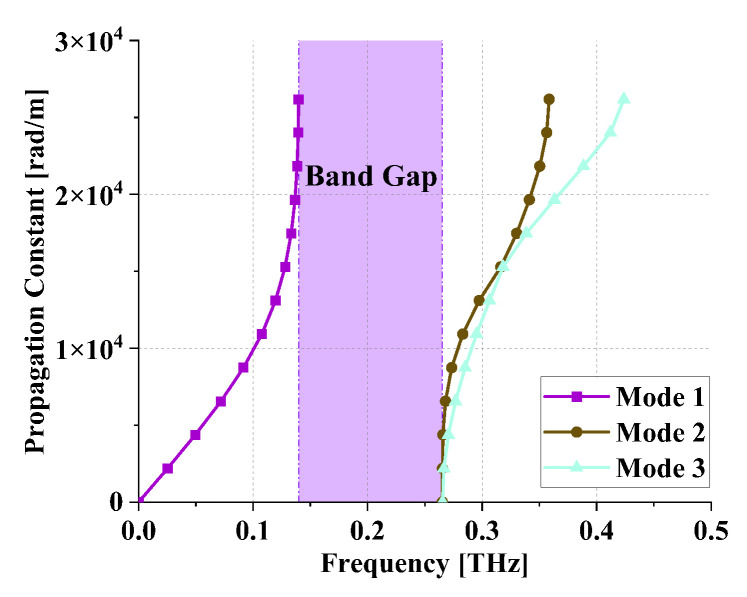
Dispersion of TSV-based EBG.

The electromagnetic simulation of the TSV-based EBG unit was conducted using CST Microwave Studio v2020 software. The dispersion is illustrated in Figure 8, revealing a central frequency of approximately 0.2 THz and a bandwidth of about 62%, aligning well with the design specifications.

**Figure 9:**
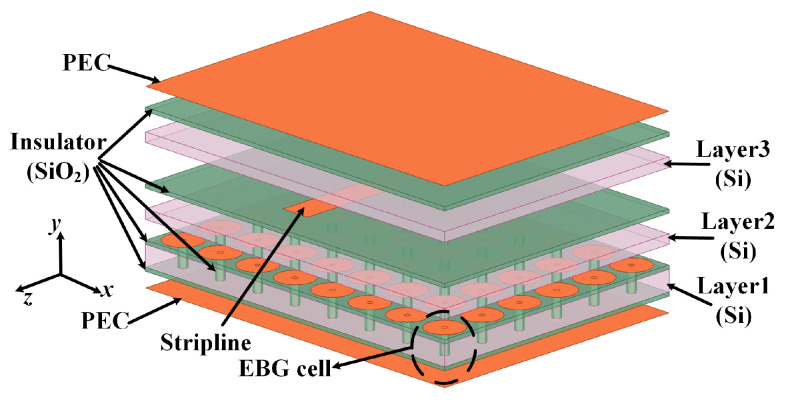
Physical structure of SSIGW.

The SSIGW is composed of stripline and TSV-based EBG array, as illustrated in Figure 9. Further optimization using the High-Frequency Structure simulator (HFSS) determines a stripline width of 20 μm. The metal stripline is etched onto the upper surface of layer SiO_2_ on the middle dielectric substrate (Layer 2, Si). The TSV-based EBG structure ensures that signal energy is confined to the stripline, minimizing energy diffusion leakage on both sides of the stripline.

**Figure 10:**
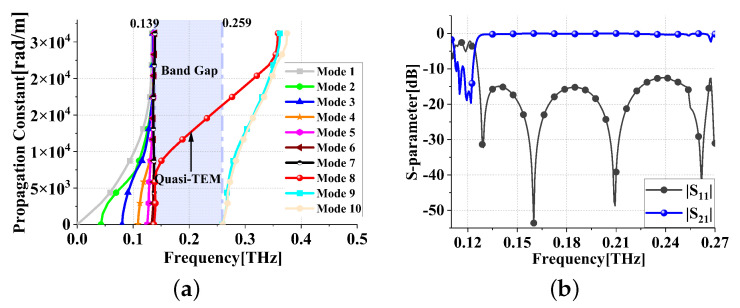
Simulation results of the SSIGW: (**a**) dispersion and (**b**) |$S_{11}$| and |$S_{21}$|.

CST Microwave Studio software is used to analyze the dispersion diagram of the SSIGW, as shown in Figure 10a. It is seen that the electromagnetic bandgap is 0.139–0.259 THz. The S parameters, |$S_{11}$| and |$S_{21}$|, of the SSIGW are shown in Figure 10b. The −10 dB bandwidth of the SSIGW starts from 0.126 THz.

### 3.2. Design and Analysis of Ultra-Wideband Bandpass Filter

**Figure 11:**
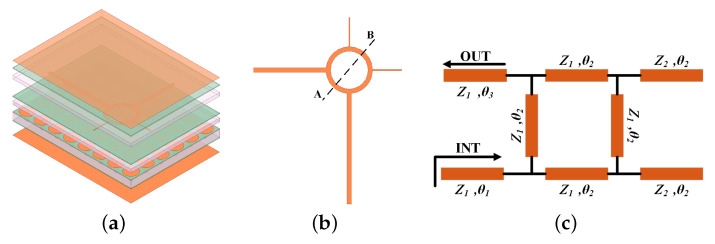
UWB bandpass filter: (**a**) 3D view; (**b**) 2D view of the resonator; and (**c**) equivalent transmission line model ($Z_{1}$ = 50 Ω, $Z_{2}$ = 100 Ω, $\theta_{1}$ = 180, $\theta_{2}$ = 90, $\theta_{3}$ = 270).

The subsection improves a UWB bandpass filter based on SSIGW in [15], and its structure is shown in Figure 11a. A non-equal-impedance branch-and-loop resonator is proposed to solve the problem of excessive in-band return loss in conventional equal-impedance branch-and-loop resonators. In Figure 11b, the schematic depicts the UWB filter utilizing a circular ring resonator loaded with a pair of quarter wavelength open circuit stubs connected to the right and top center of the ring. The input and output ports are positioned at two orthogonal locations, specifically at the bottom and left center of the ring, and are directly linked to the ring. The equivalent transmission line model is illustrated in Figure 11c. The circumference of the ring is one wavelength, and the characteristic impedance is $Z_{1}$, while the electrical length of the open circuit stub is $\theta_{2}$ and the characteristic impedance is $Z_{2}$.

**Figure 12:**
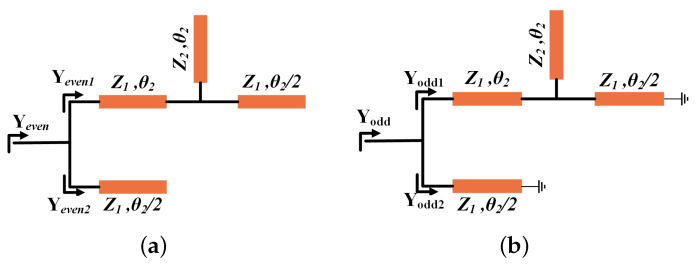
Even and odd mode analysis: (**a**) even mode transmission line model; (**b**) odd mode transmission line model.

Under weak coupling [25,26], the plane of symmetry AB shown in Figure 11b can be considered as an ideal magnetic or electrical wall, respectively. The equivalent even/odd mode transmission line model of the ring resonator used to determine the resonant frequency of the filter is shown in Figure 12a and Figure 12b, respectively.

According to the ring resonator theory, it can be concluded that the ring resonator resonates when its input admittance is zero [25]. Therefore, the resonance conditions are shown as follows:

(10) $$Y_{even}=Y_{even1}+Y_{even2}=0$$

(11) $$Y_{odd}=Y_{odd1}+Y_{odd2}=0$$

In this subsection, the odd–even mode method is used to calculate the resonance frequency of a circular ring resonator loaded with an open circuit stub. The even mode equivalent transmission line model when a magnetic wall is applied to the symmetric plane AB of the ring resonator is shown in Figure 12a. The analysis is simplified by neglecting all effects of transmission line discontinuities within the ring resonator depicted in Figure 11b. The normalized even mode input admittances $Y_{even1}$ and $Y_{even2}$ of the upper and lower arms are, respectively, expressed by the following formula:

(12) $$Y_{even1}=j\frac{-m^{3}-Knm^{2}+3m+Kn}{-3m^{2}-2Knm+1}$$

(13) $$Y_{even2}=jm$$

where $m=\tan\left(\theta_{2}/2\right)$, $n=\tan\theta_{2}=2m/\left(1-m^{2}\right)$, $K=Z_{1}/Z_{2}$.

Thus, the even mode resonance condition (10) can be obtained by the following equation:

(14) $$Y_{even}=-j\frac{4m^{3}+3Knm^{2}-4m-Kn}{-3m^{2}-2Knm+1}=0$$

Odd mode analysis is akin to even mode. In Figure 12b, the equivalent transmission line model represents a half-ring resonator with an electrical wall applied along AB. The normalized odd mode input admittances $Y_{odd1}$ and $Y_{odd2}$ of the upper and lower arms can be deduced as follows:

(15) $$Y_{odd1}=-j\frac{Knm^{3}-3m^{2}-Kmn+1}{-m^{3}-2Knm^{2}+3m}$$

(16) $$Y_{odd2}=1/jm$$

Therefore, the odd mode resonance condition (11) can be obtained by the following equation:

(17) $$Y_{odd}=j\frac{Knm^{3}-4m^{2}-3Knm+4}{m^{3}+2Knm^{2}-3m}=0$$

If the loop is assumed to be lossless, the frequency at which the transmission zero is located can be obtained as shown in [26], by solving the following:

(18) $$Y_{even}=Y_{odd}$$

By substituting (14) and (17) into (18), the following polynomial equation can be obtained.

(19) $$\begin{array}{cc} \; & m^{6}+2Knm^{5}+\left(K^{2}n^{2}-1\right)m^{4}+\left(K^{2}n^{2}-1\right)m^{2}-2Knm+1=0 \end{array}$$

The resonant frequency can be calculated by the following formula:

(20) $$f_{r}=\frac{2\pi c}{\theta_{2}\lambda_{g}\sqrt{\varepsilon_{eff_{2}}}}$$

where *c* is the speed of light in free space, and $\lambda_{g}$ is the waveguide wavelength. This excerpt uses K=1/2, $\lambda_{g}=0.61$ mm, $\varepsilon_{eff_{2}}=10.583$.

Table 2 shows the comparison between the calculated and simulated resonance frequencies. It can be seen that the calculated values agree with the simulations. The causes of errors include loss of loops, discontinuity, and so on. In addition, the calculation has only two transmission zeros, while the simulation has four. The additional transmission zeros are generated by SSIGW itself, as can be seen from Figure 10b.

**Figure 13:**
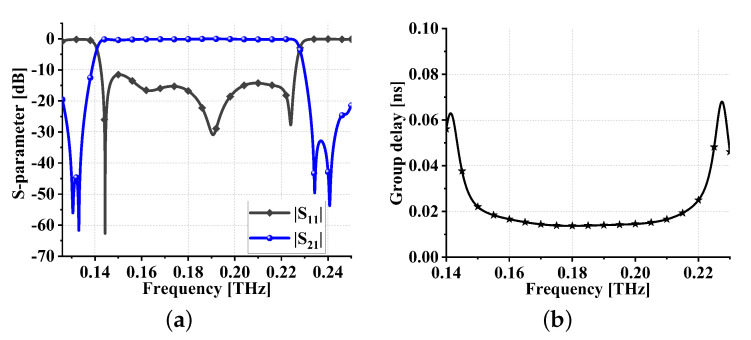
Simulation results of UWB bandpass filter: (**a**) S parameters and (**b**) in-band group delay.

Simulated |$S_{11}$| and |$S_{21}$| are shown in Figure 13a. As can be seen from Figure 13a, its passband is 0.142–0.226 THz, and its fractional bandwidth (FBW) is 45.34% around the center frequency of 0.184 THz. The insertion loss is lower than 0.71 dB, and the out-of-band suppression is higher than 20 dB. Simulated group delay is shown in Figure 13b. As can be seen from Figure 13b, its in-band group delay is 0.037 ± 0.023 ns, very flat over the bandwidth.

### 3.3. Design and Analysis of Meta-Surface Wideband Bandstop Filter

**Figure 14:**
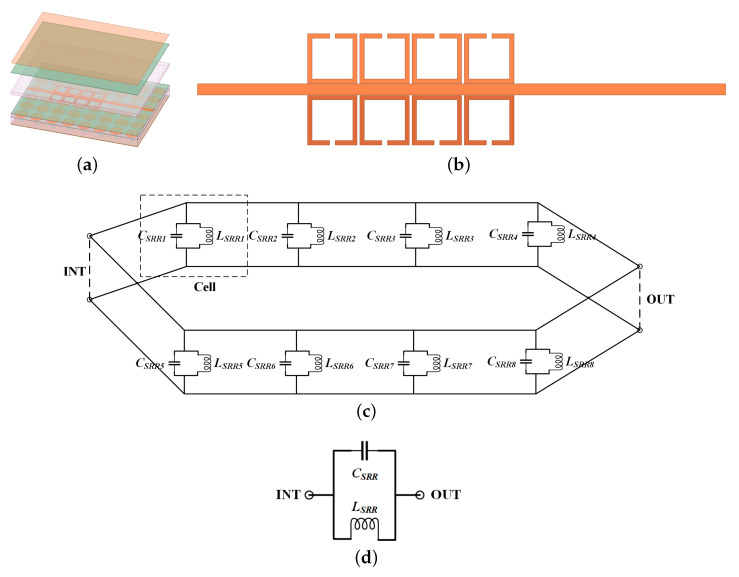
Meta-surface bandstop filter: (**a**) 3D view; (**b**) 2D view of the filter resonator; (**c**) the equivalent circuit; and (**d**) the circuit unit.

This subsection introduces a novel meta-surface bandstop filter, depicted in Figure 14a. The stopband functionality is achieved by adding SRRs on both sides of the transmission line. Adjusting the dimensions of the SRRs allows for the realization of stopbands in different frequency ranges.

**Figure 15:**
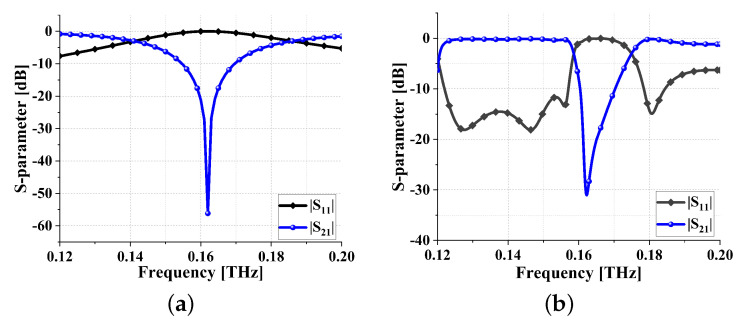
S parameters of filter: (**a**) equivalent circuit; (**b**) meta-surface bandstop filter 1.

The planar view and corresponding equivalent circuit are depicted in Figure 14b and Figure 14c, respectively. Figure 14d illustrates the circuit unit. To elucidate the operational principles, a detailed analysis of the equivalent circuit unit follows. Empirical formulas (21)–(24) are derived from both [27,28] and the SSIGW physical model presented in this paper. The calculation of the dimensions of meta-surface bandstop filter 1 yields $L_{SRR}$ = 0.0272 nH, $C_{SRR}$ = 0.033 pF. Following optimization using the Advanced Design System (ADS), the parameters are confirmed to be $L_{SRR}$ = 0.0272 nH, $C_{SRR}$ = 0.035 pF, and the S-parameter is displayed in Figure 15a. From Figure 15b, it can be observed that the resonant points from theoretical calculations and simulations coincide at 0.162 THz.

(21) $$L_{SRR}=\frac{\mu_{r}\mu_{0}\ln(1+32{\left(8h_{3}+T\right)}^{2}\left(1+\sqrt{1+\pi w_{M1}^{2}/\left(8h_{3}+T\right)}/w_{M1}^{2}\right)}{100000\pi }$$

(22) $$C_{SRR}=\frac{\varepsilon_{eff2}\varepsilon_{0}M}{9000}$$

(23) $$M=\frac{2\ln(2\left(1+\sqrt{K}\right)/\left(1-\sqrt{K}\right))}{\pi }$$

(24) $$K=\sqrt{1-\frac{1}{{\left(1+2w_{M1}/S_{M1}\right)}^{2}}}$$

in which $\varepsilon_{eff2}=10.583$ represents the relative permittivity, $\mu_{r}=1$ is the relative magnetic permeability, $w_{M1}$ = 8 μm is the metal line width of the SRR, and $S_{M1}$ = 8 μm is the gap distance between SRR openings.

**Figure 16:**
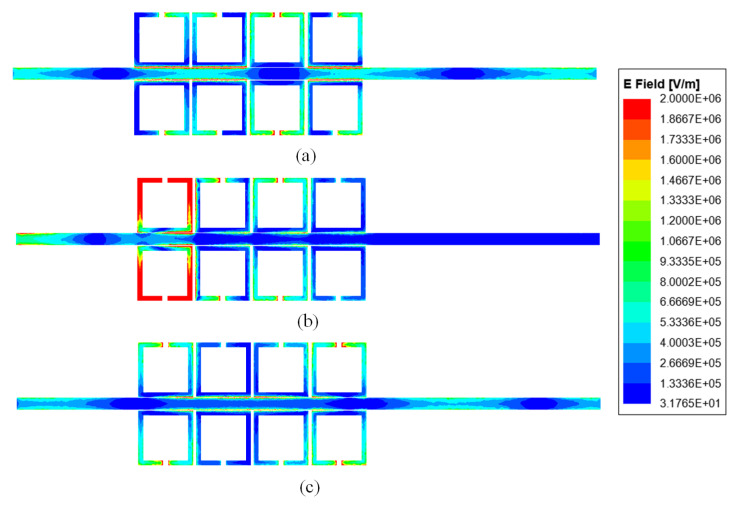
Electric field profile of meta-surface bandstop filter 1: (**a**) outside the stopband (@0.14 THz); (**b**) in-band (@0.165 THz); and (**c**) outside the stopband (@0.18 THz).

As can be seen from Figure 16a, inside the stopband (@0.165 THz), energy is absorbed by meta-surface losses, especially on the first unit. As can be seen from Figure 16b,c, outside the stopband (@0.14 THz and @0.18 THz), energy concentrates on the transmission line and is propagated to the output end. This further validates the accuracy of the theoretical analysis mentioned above.

**Figure 17:**
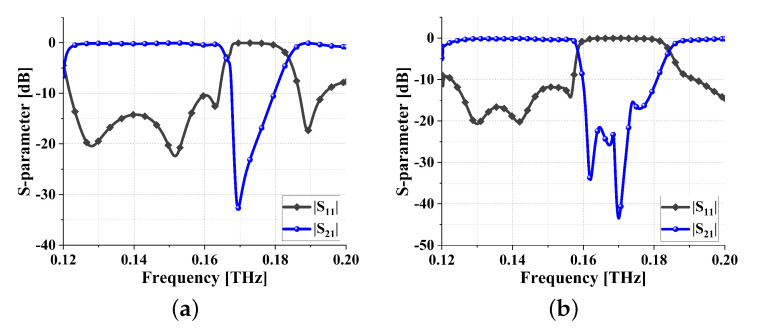
S parameters of filters: (**a**) meta-surface bandstop filter 2; and (**b**) wide stopband meta-surface bandstop filter.

In order to broaden the bandwidth, this paper designed meta-surface bandstop filter 2 with adjacent stopband ranges. The S-parameters, as shown in Figure 17a, reveal a stopband range of 0.168–0.18 THz. Overlaying the two configurations resulted in a meta-surface filter with a wide stopband, as depicted in Figure 17b, now exhibiting a stopband range of 0.16–0.181 THz. This configuration effectively satisfies the criteria for broad stopband performance.

### 3.4. Results and Comparisons

**Figure 18:**
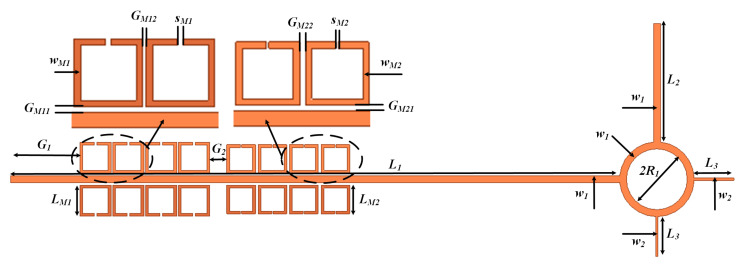
Parameters of meta-surface THz bandpass filter.

**Figure 19:**
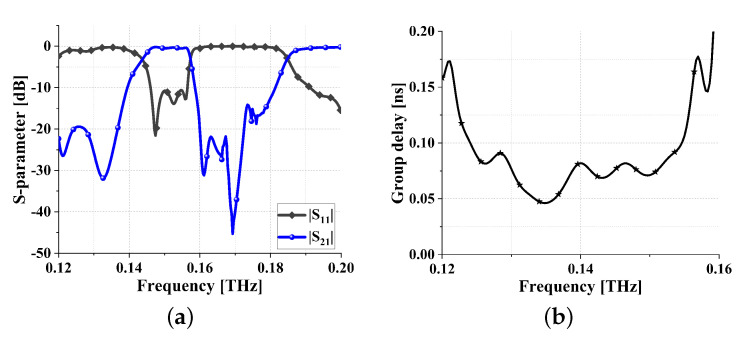
Simulation results of meta-surface THz bandpass filter: (**a**) S parameters and (**b**) in-band group delay.

The parameters and their corresponding values for the filter proposed in this paper, after the final optimization, are presented in Table 3. The specific physical description of these parameters is illustrated in Figure 18. The simulation results of the proposed THz filter are shown in Figure 19. From Figure 19a, it can be observed that the center frequency is 0.151 THz, the FBW is 6.9%, the insertion loss is less than 0.68 dB, and both the upper and lower stopband widths exceed 20 GHz. Simulated group delay is shown in Figure 19b. As can be seen from Figure 19b, the in-band group delay is 0.119 ± 0.048 ns, again very flat.

**Figure 20:**
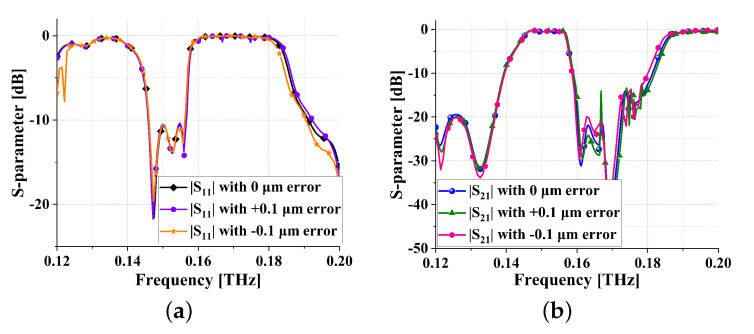
Error simulation results of meta-surface THz bandpass filter: (**a**) |$S_{11}$| and (**b**) |$S_{21}$|.

Due to the influence of processing errors on the performance of filters, and considering that the processing accuracy of TSV can reach the nanometer level, this subsection simulates the filter with an error of ±0.1 μm. The simulation results are shown in Figure 20, where Figure 20a represents the |$S_{11}$| with a ±0.1 μm error, and Figure 20b represents the |$S_{21}$| with a ±0.1 μm error. It can be observed that the actual processing errors of the filter designed in this study have almost no impact, demonstrating high stability. Furthermore, the operating temperature can also contribute to changes in the performance of the filter. In this study, TSV technology is employed, and the impact of the operating temperature on the filter is determined by the TSV operating temperature. According to [29,30], it can be concluded that the stable operating temperature range for the filter proposed in this paper is from −40 °C to 120 °C.

**Figure 21:**
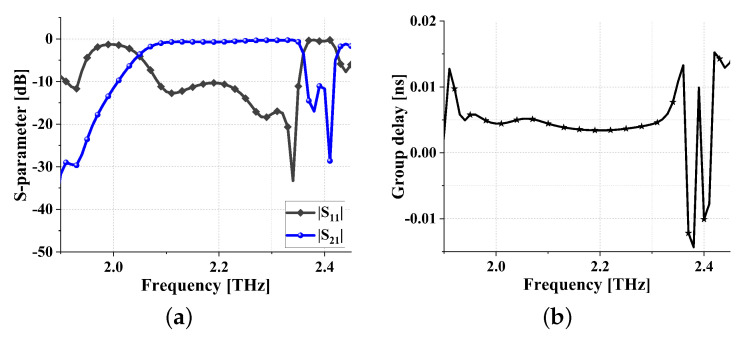
Simulation results of another meta-surface THz bandpass filter: (**a**) S parameters and (**b**) in-band group delay.

To further demonstrate the universality of the theoretical framework proposed in this article, this section presents the filtering results in another frequency band, as shown in Figure 21. From Figure 21a, it can be observed that the center frequency is 2.215 THz, the FBW is 6.1%, the insertion loss is less than 1.29 dB, and both the upper and lower stopband widths exceed 50 GHz. Simulated group delay is shown in Figure 21b. As can be seen from Figure 21b, the in-band group delay is 0.007 ± 0.004 ns, again very flat.

A comparison of the above designed filters with the reported six related filters is performed in Table 4. Note that the SSIGW filter showcases ultra-low latency and exceptional filtering characteristics. In comparison to the existing literature [3,4], the work demonstrates smaller insertion losses and is more easily integrable than filters designed using SIW, as SSIGW supports TEM mode. Unlike previous studies, the work excels in achieving lower insertion losses than filters designed using other techniques. Additionally, the proposed filter exhibits a 21-fold reduction in group delay compared to [4], making it more suitable for low-latency communication in the THz range.

## 4. Conclusions

THz filters currently face challenges such as significant losses, integration difficulties, and complexities in designing filters with arbitrary bandwidths. This paper proposes a meta-surface-based approach for designing arbitrary bandwidth SSIGW filters, effectively addressing the aforementioned challenges. The method achieves bandwidth control by incorporating a meta-surface-based bandstop filter into a UWB filter and controlling the operating frequency range of the meta-surface bandstop filter. Additionally, the utilization of SSIGW enables functionalities such as low loss and easy integration. To validate the effectiveness and superiority of the proposed method, a THz filter is designed in this study. In the UWB filtering section, non-equal-impedance branch-and-loop resonator is employed, and the analysis is conducted using transmission line theory, with the theoretical and simulated results aligning closely. The bandstop filtering section utilizes SRR meta-surfaces, analyzed through an equivalent circuit analysis, with the theoretical and simulated results aligning closely. Adjusting the operating frequency range of the SRR meta-surface allows for different bandwidths, while increasing the number of SRRs enables a wider stopband. The presented filter exhibits an insertion loss below 0.68 dB, with a stopband width exceeding 20 GHz and a group delay of 0.119 ± 0.048 ns. Compared to existing THz filters, it exhibits advantages such as lower loss, lower group delay, and controllable arbitrary bandwidth. Therefore, it is expected to become a candidate for THz wireless communication systems.

## Figures and Tables

**Table 1:** Dimensions of TSV-based EBG (units: μm).

*T*	*p*	*r*	*R*	$h_{1}$	$h_{2}$	$h_{3}$
5	120	7	50	55	25	25

**Table 2:** Comparison of calculated and simulated resonant frequencies (units: THz).

Resonant Frequency	Calculated	Simulated
TZ1	0.128	0.130
TZ2	–	0.133
TZ3	0.224	0.234
TZ4	–	0.241
TP1	0.151	0.144
TP2	0.191	0.191
TP3	0.220	0.224

**Table 3:** Dimensions of meta-surface THz bandpass filter (units: μm).

Parameters	Values	Parameters	Values	Parameters	Values
$L_{1}$	1738	$L_{2}$	352	$L_{3}$	125
$R_{1}$	98	$w_{1}$	20	$w_{2}$	7
$L_{M1}$	90	$S_{M1}$	8	$w_{M1}$	8
$L_{M2}$	83	$S_{M2}$	3	$w_{M2}$	8
$G_{M11}$	9	$G_{M12}$	5	$G_{1}$	200
$G_{M21}$	9	$G_{M22}$	8	$G_{1}$	50

**Table 4:** Comparison with related THz filters.

Filters	Type	Method	Center Frequency [THz]	FBW [%]	Insertion Loss [dB]	Group Delay [ns]
						
[1]	Meta-material	Sim.	7	22.85	- -	3.67
[2]	Meta-material	Sim.	0.125	65	- -	- -
[3]	SIW	Meas.	0.331	15.4	1.5	- -
[4]	SIW	Sim.	0.160	12.5	1.5	2.5 ± 1.5
[13]	Hairpin	Sim.	0.500	16	1.5	- -
[31]	CNC milling	Meas.	0.292	14.64	3	- -
[32]	Meta-material	Sim.	1	18	3	- -
[33]	Meta-material	Meas.	0.6	- -	>3	- -
[34]	Meta-material	Sim.	2.37	59	3	- -
This work 1	SSIGW	Sim.	0.151	6.9	0.68	0.119 ± 0.048
This work 2	SSIGW	Sim.	2.215	6.1	1.29	0.007 ± 0.004

## Data Availability

Data are contained within the article.
